# Supplementary material for: Action execution and action observation elicit mirror responses with the same temporal profile in human SII
Source: Commun Biol. 2020 Feb 20;3:80. doi: 10.1038/s42003-020-0793-8 (PMC7033229; doi:10.1038/s42003-020-0793-8)
Supplement: Supplementary file 1 — Supplementary Information [file 42003_2020_793_MOESM1_ESM.docx]

**Supplementary material**

**
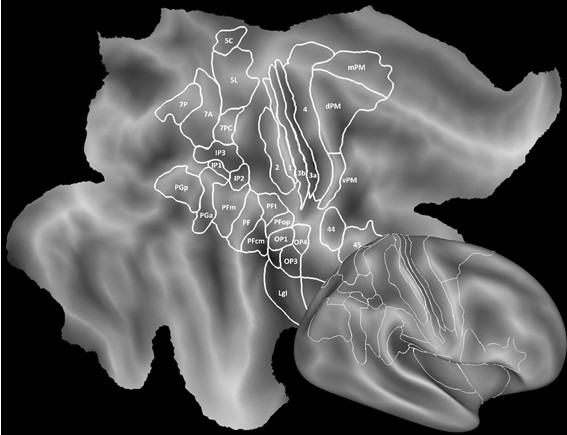
**

**Supplementary Figure 1: Brain template.** Flat map of fs-LR-average right hemisphere template (163.842 nodes), with cytoarchitectonic regions indicated. The bottom-right inset shows the inflated view of the same brain template. Same conventions are used for the brain insets shown in Figure 2 and 3.

**
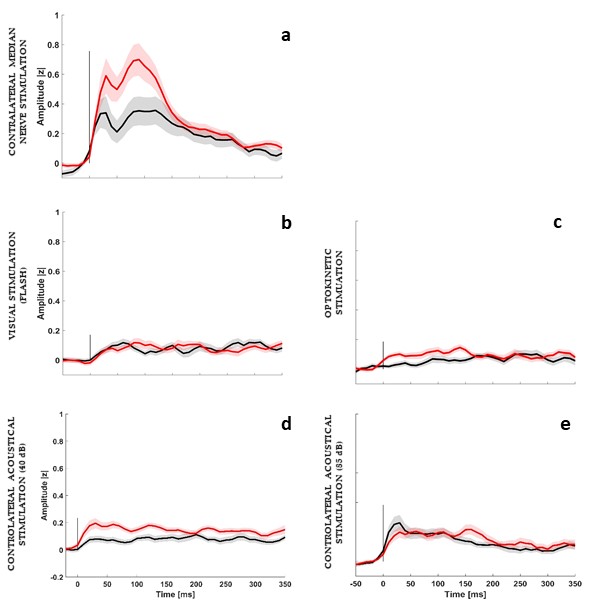
**

**Supplementary Figure 2: Gamma band time-course following neurophysiological tests of SII leads responsive in at least one phase of the experimental paradigm.** Figure 2 shows the normalized (z-score) gamma band temporal course (±SE) of both left (red trace) and right SII (black trace) in response to median nerve (panel a), to visual (panel b), to optokinetic (panel c), to acoustical stimulation (panel d and e, respectively to 40 and 85 dB SPL). The average has been computed taking into account all leads (22 left, 19 right) with a statistically significant response to at least one phase of the experimental paradigm (*see Methods*). For visualization purposes, data are Savitzky-Golay filtered.

**
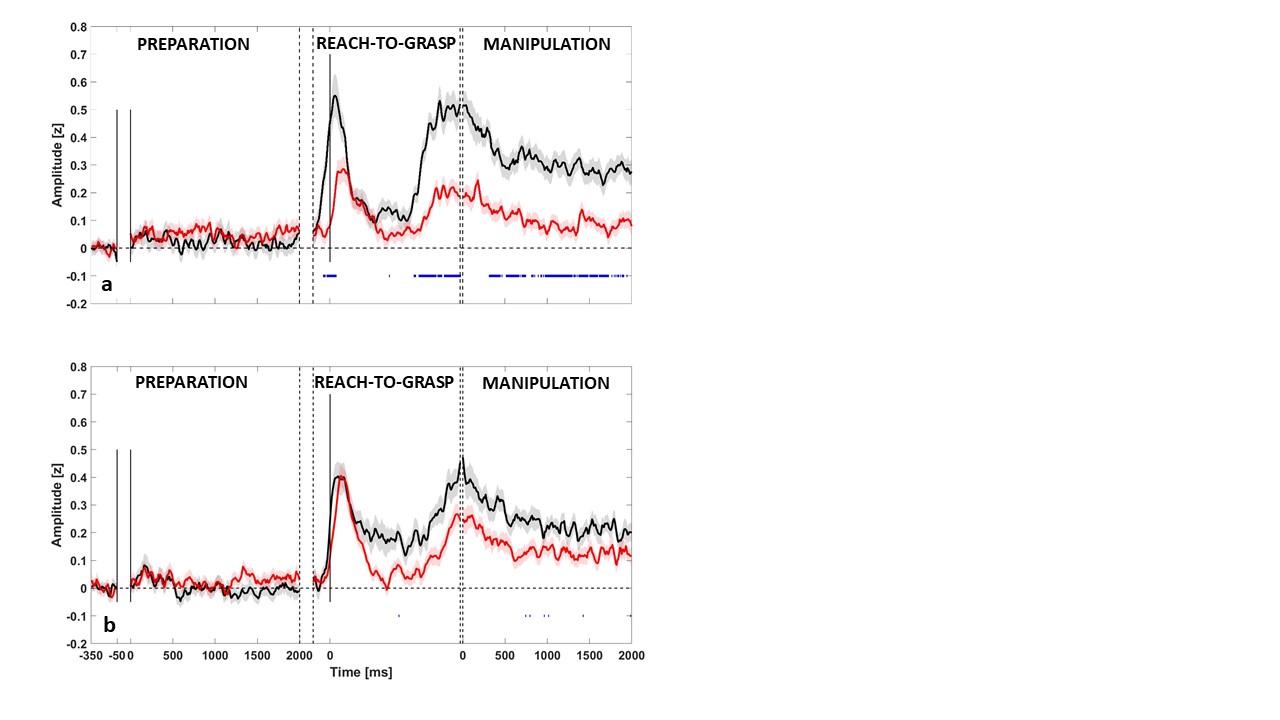
Supplementary Figure 3: Statistical comparison between left and right SII gamma responses to action execution and observation**. The figure depicts for left SII (panel a) and right SII (panel b) the average time-course (±SE) for execution (black trace) and observation condition (red trace). The averaged amplitude is computed in terms of z-score respect to the baseline for each trial *(see Methods*) including all leads responsive in at least one phase (22 left, 19 right). Significance between the two conditions is corrected with Bonferroni for each phase and reported below the traces. For visualization purposes, data are Savitzky-Golay filtered.

**
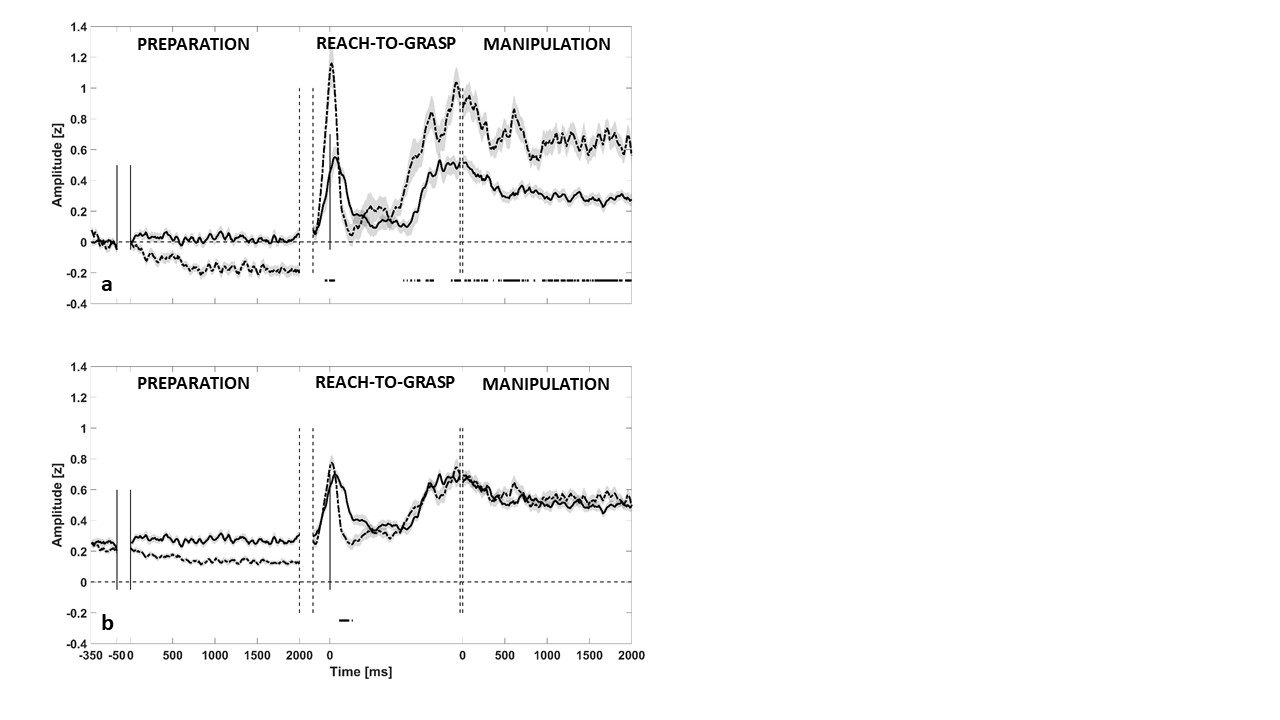
** **Supplementary Figure 4: Statistical comparison between contralateral SI and SII responses to action execution**. The figure depicts the statistical comparison between contralateral SI (dotted line) and SII. The average amplitude (panel a) is computed in terms of z-score (±SE) respect to the baseline for each trial (*see Methods*) including all leads in at least one phase (22 left SII, 12 left SI). Furthermore, panel b presents the same data, after normalization per leads in the range of amplitude 0-1. Statistical significance (Bonferroni corrected) for reach-to-grasp and manipulation phases is shown below the traces in each panel. For visualization purposes, data are Savitzky-Golay filtered.

**
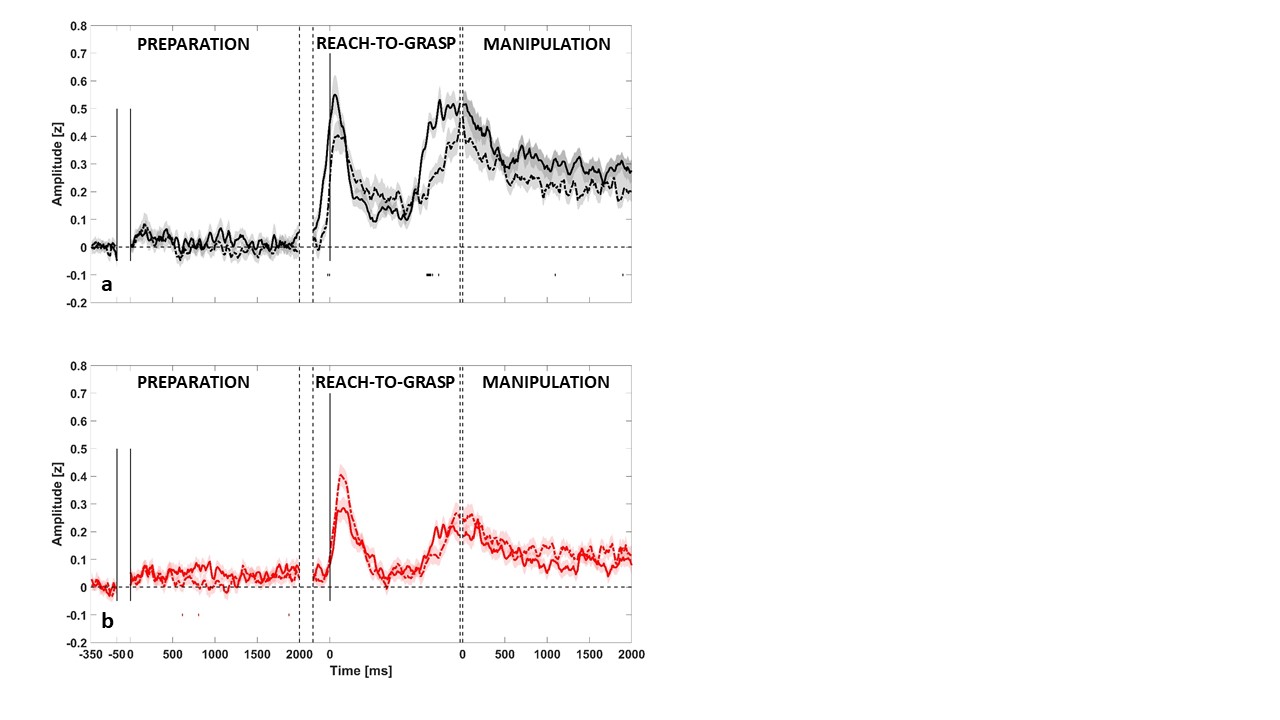
Supplementary Figure 5: Statistical comparison between contralateral and ipsilateral SII in execution and observation.** The figure depicts the statistical comparison between contralateral SII and ipsilateral SII (dotted line) during execution (panel A) and observation (panel B). In both cases, the averaged amplitude is computed in terms of z-score (±SE) respect to the baseline for each trial (*see Methods*) including all leads in at least one phase (22 left, 19 right). Statistical significance is corrected with Bonferroni for each phase and shown below the traces in each panel. For visualization purposes, data are Savitzky-Golay filtered.

| **Test** | ***t range*** | | | ***minimum p-value***  ***(Bonferroni corrected)*** | | | ***dof*** | ***effect size (Cohen’s d)*** | | |
| --- | --- | --- | --- | --- | --- | --- | --- | --- | --- | --- |
|  | **PREP** | **RTG** | **MANIP** | **PREP** | **RTG** | **MANIP** |  | **PREP** | **RTG** | **MANIP** |
| **Contralateral SII, execution (against the baseline)** | **[-2.57, 3.13]** | **[0.88, 15.62]** | **[5.67, 16.12]** | **1.02** | **< 0.001** | **< 0.001** | **21** | **[-0.55, 0.67]** | **[0.19, 3.32]** | **[1.21, 3.44]** |
| **Ipsilateral SII, execution (against the baseline)** | **[-3.91, 3.32]** | **[-1.27, 11.39]** | **[3.74, 12.35]** | **0.20** | **< 0.001** | **< 0.001** | **18** | **[-0.9, 0.76]** | **[-0.29, 2.61]** | **[0.86, 2.83]** |
| **Contralateral SII, observation (against the baseline)** | **[-1.65, 7.75]** | **[0.31, 10.27]** | **[0.63, 8.37]** | **< 0.001** | **< 0.001** | **< 0.001** | **21** | **[-0.35, 1.65]** | **[0.07, 2.19]** | **[0.14, 1.78]** |
| **Ipsilateral SII, observation (against the baseline)** | **[-4.23, 5.06]** | **[-0.98, 12.73]** | **[1.84, 12.13]** | **0.07** | **< 0.001** | **< 0.001** | **18** | **[-0.97, 1.16]** | **[-0.23, 2.93]** | **[0.42, 2.78]** |
| **Contralateral SII (execution vs observation)** | **[-3.76, 2.52]** | **[-1.08, 8.24]** | **[-2.05, 8.42]** | **0.10** | **< 0.001** | **< 0.001** | **41** | **[-1.13, 0.69]** | **[-0.32, 2.49]** | **[0.66, 2.45]** |
| **Ipsilateral SII (execution vs observation)** | **[-4.06, -3.02]** | **[-2.91, 4.07]** | **[-2.66, 4.93]** | **0.05** | **< 0.001** | **< 0.001** | **37** | **[-1.32, 0.84]** | **[-0.94, 1.32]** | **[-0.39, 1.6]** |

**Supplementary Table 1:** Supplementary Table 1 reports, for each time-wised test, the correspondent t- values range, the minimum p value, the degrees of freedom and the effect size (*Cohen’s d*). PREP: preparation, RTG: reaching-to-grasp, MANIP: manipulation.
