# Supplementary material for: Action execution and action observation elicit mirror responses with the same temporal profile in human SII
Source: Commun Biol. 2020 Feb 20;3:80. doi: 10.1038/s42003-020-0793-8 (PMC7033229; doi:10.1038/s42003-020-0793-8)
Supplement: Supplementary file 2 — Reporting Summary [file 42003_2020_793_MOESM2_ESM.pdf]

nature research

Corresponding author(s): MARIA DEL VECCHIO

Last updated by author(s): Jan 9, 2020

Reporting Summary

Nature Research wishes to improve the reproducibility of the work that we publish. This form provides structure for consistency and transparency in reporting. For further information on Nature Research policies, see Authors & Referees and the Editorial Policy Checklist.

Statistics

For all statistical analyses, confirm that the following items are present in the figure legend, table legend, main text, or Methods section.

n/a	Confirmed	
☐	☒	The exact sample size (*n*) for each experimental group/condition, given as a discrete number and unit of measurement
☐	☒	A statement on whether measurements were taken from distinct samples or whether the same sample was measured repeatedly
☐	☒	The statistical test(s) used AND whether they are one- or two-sided<br>*Only common tests should be described solely by name; describe more complex techniques in the Methods section.*
☒	☐	A description of all covariates tested
☐	☒	A description of any assumptions or corrections, such as tests of normality and adjustment for multiple comparisons
☐	☒	A full description of the statistical parameters including central tendency (e.g. means) or other basic estimates (e.g. regression coefficient) AND variation (e.g. standard deviation) or associated estimates of uncertainty (e.g. confidence intervals)
☐	☒	For null hypothesis testing, the test statistic (e.g. *F*, *t*, *r*) with confidence intervals, effect sizes, degrees of freedom and *P* value noted<br>*Give P values as exact values whenever suitable.*
☒	☐	For Bayesian analysis, information on the choice of priors and Markov chain Monte Carlo settings
☒	☐	For hierarchical and complex designs, identification of the appropriate level for tests and full reporting of outcomes
☒	☐	Estimates of effect sizes (e.g. Cohen's *d*, Pearson's *r*), indicating how they were calculated

Our web collection on statistics for biologists contains articles on many of the points above.

Software and code

Policy information about availability of computer code

Data collection	sEEG data were collected with Nihon Khoden EEG data acquisition software
Data analysis	anlaysis was performed with custom made Matlab code, available upon request

For manuscripts utilizing custom algorithms or software that are central to the research but not yet described in published literature, software must be made available to editors/reviewers. We strongly encourage code deposition in a community repository (e.g. GitHub). See the Nature Research guidelines for submitting code & software for further information.

Data

Policy information about availability of data

All manuscripts must include a data availability statement. This statement should provide the following information, where applicable:

- Accession codes, unique identifiers, or web links for publicly available datasets
- A list of figures that have associated raw data
- A description of any restrictions on data availability

All the data supporting the findings of the study are available from the corresponding author upon request

Field-specific reporting

Please select the one below that is the best fit for your research. If you are not sure, read the appropriate sections before making your selection.

☒ Life sciences ☐ Behavioural & social sciences ☐ Ecological, evolutionary & environmental sciences

For a reference copy of the document with all sections, see nature.com/documents/nr-reporting-summary-flat.pdf

Life sciences study design

All studies must disclose on these points even when the disclosure is negative.

Sample size	Sample size was decided from the coverage of the primary and secondary somatosensory cortex and this led to include 18 patients.
Data exclusions	No data were excluded.
Replication	We did not replicate the results in a separte cohort of patients; However short trial data can be considered as a replication for the purpose of most of the analyses.
Randomization	Randomization of participants was not relevant to the study, and each subject completed an experimental session in which order of tests was fixed. However within a block the order of trials was randomized.
Blinding	Blinding was not relevant to the study. All participants performed the same tasks.

Reporting for specific materials, systems and methods

We require information from authors about some types of materials, experimental systems and methods used in many studies. Here, indicate whether each material, system or method listed is relevant to your study. If you are not sure if a list item applies to your research, read the appropriate section before selecting a response.

Materials & experimental systems

n/a	Involved in the study	
☒	☐	Antibodies
☒	☐	Eukaryotic cell lines
☒	☐	Palaeontology
☒	☐	Animals and other organisms
☐	☒	Human research participants
☒	☐	Clinical data

Methods

n/a	Involved in the study	
☒	☐	ChIP-seq
☒	☐	Flow cytometry
☒	☐	MRI-based neuroimaging

Human research participants

Policy information about studies involving human research participants

Population characteristics	Data were collected from 18 patients (12 males, 6 females) suffering from drug-resistant focal epilepsy (age 36±7)
Recruitment	Each patient who underwent an implantation for diagnostic purposes was asked for his willingness to participate in the study
Ethics oversight	The present study received the approval of the Ethics Committee of Ospedale Ca'Granda-Niguarda (ID 939-2.12.2013).

Note that full information on the approval of the study protocol must also be provided in the manuscript.
